# Evidence for Clonally Associated Increasing Rates of Azithromycin Resistant* Neisseria gonorrhoeae* in Rio de Janeiro, Brazil

**DOI:** 10.1155/2019/3180580

**Published:** 2019-01-20

**Authors:** Késia T. Barros dos Santos, Larissa B. Skaf, Livia H. Justo-da-Silva, Raphael C. Medeiros, Ronaldo da S. Francisco Junior, Maria Cristina A. Caniné, Sergio E. L. Fracalanzza, Raquel R. Bonelli

**Affiliations:** ^1^Instituto de Microbiologia Paulo de Góes, Universidade Federal do Rio de Janeiro, Rio de Janeiro 21941-902, Brazil; ^2^Laboratório Nacional de Computação Científica, Petrópolis, 26651-075, Brazil; ^3^DASA-Rua Xavier Pinheiro, 439, Parque Duque, 25085-007, Duque de Caxias, RJ, Brazil

## Abstract

Azithromycin is one of the drugs used in the combined therapy for syndromic treatment of gonorrhoea in many countries, including Brazil. Our research group, which receives isolates from clinical laboratories since 2006, has detected, after 2016, a tendency of rising rates of azithromycin resistance, with isolates showing higher minimal inhibitory concentrations (MICs) than those previously reported in this country. In this study, we report the susceptibility to azithromycin of 93* N. gonorrhoeae* isolates obtained between 2014 and 2017. Strains with MIC ≥2 *μ*g/mL were characterized according to azithromycin resistance mechanisms and strain typing. Results indicate that azithromycin resistance has emerged in all these years in unrelated MLST-STs, but after 2016 a clonal complex connected with ST1901 has been more frequently detected, grouping isolates with MIC varying from 2 to 64 *μ*g/mL, with DelA mutations at the* mtrR* promoter region associated or not with mutations at* rrl* alleles. High rates of azithromycin resistance may compromise the use of this drug in the combined therapy with ceftriaxone. Inclusion of Rio de Janeiro in the Brazilian gonococcal surveillance program is important to evaluate if this data indicates an epidemiological phenomenon in the country.

## 1. Introduction

*Neisseria gonorrhoeae*, the agent of gonorrhoea, has come to world attention due to a high capacity to become resistant to antimicrobial agents [1, 2]. Currently, empiric treatment for gonorrhoea is based on ceftriaxone combined with azithromycin in most countries [3–5]. Combined therapy is a strategy to mitigate the dissemination of resistant strains to any of the drugs used [6]. In many countries, including Brazil, monotherapy with 2 g azithromycin is used to treat gonorrhoea in patients allergic to beta lactams [3, 7].

The minimal inhibitory concentration (MIC) breakpoint that defines azithromycin resistance in* N. gonorrhoeae* is not under consensus. Whereas EUCAST classifies isolates with MIC >0.5 *μ*g/mL as resistant to azithromycin, CLSI indicates the epidemiological cutoff value (ECV) ≥2 *μ*g/mL as an alert value to recognize isolates carrying resistance mechanisms to this drug [8, 9]. Different molecular mechanisms may cumulatively reduce the susceptibility to azithromycin in* N. gonorrhoeae*, including Erm methylases, mutations in genes encoding ribosomal proteins L4 and L22, and mutations that affect the expression or identity of genes encoding the efflux pump MtrCDE [1, 10]. However, mutations in one to four alleles of* rrl*, which encodes the target of azithromycin 23S rRNA, usually exert greater impact in the azithromycin MIC, in some cases reaching 256 *μ*g/mL [10–12].

Bazzo et al. [13] have recently published the results of a Brazilian national gonococcal antimicrobial surveillance program (GASP), based on the analysis of 550 isolates collected during 2015 and 2016 in seven sentinel sites located in five geographical regions of the country. Data indicated high rates of ciprofloxacin, tetracycline and penicillin resistance, and full susceptibility to cefixime and ceftriaxone. Regarding azithromycin, 6.9% of the isolates were considered resistant according to EUCAST breakpoints, and 1.3% of the isolates had MIC ≥ 2 *μ*g/mL [13]. Rio de Janeiro was not included in the Brazilian-GASP. However, we performed a study based on isolates obtained from 2006 to 2015 in this city indicating 17% azithromycin resistance, based on CLSI ECV [14]. In that study, isolates were recovered mainly from private clinical laboratories at their convenience, but without any screening [14]. In the present study, we describe the increase of azithromycin resistance rate and azithromycin MIC among* N. gonorrhoeae* isolates received by our research laboratory from 2014 to 2017. Strain typing indicates the emergence of a lineage belonging to a new multilocus sequence type (ST) with MIC higher than those previously reported in Brazil [13, 14].

## 2. Materials and Methods

### 2.1. Sampling

Between March 2014 and October 2017, 93* N. gonorrhoeae* isolates were sent by public healthcare facilities and private clinical laboratories to our Laboratory of Investigation in Medical Microbiology (LIMM) at the Universidade Federal do Rio de Janeiro. No criteria have been applied for the selection of isolates. At LIMM, isolates were identified by MALDI-TOF MS (Bruker Biotyper 3.1, Bruker Daltonics, Germany) and those confirmed as* N. gonorrhoeae* were preserved in BHI broth supplemented with 20% glycerol at -170°C. Specimen types were urethral (n= 70), urine (n =14), vaginal (n = 7), eye discharge (n=1), and rectal (n = 1) types. Eighty-five patients were males and eight were females. Patient ages varied between 16 and 70 years (median 29). Patient sexual orientation is unknown.

### 2.2. Antimicrobial Susceptibility Testing

Antimicrobial susceptibility testing was performed with discs containing penicillin (10 U), tetracycline (30 *μ*g), ciprofloxacin (5 *μ*g), or ceftriaxone (30 *μ*g). Azithromycin (Sigma) MIC was determined by agar dilution.* N. gonorrhoeae* ATCC 49226 was used as quality control strain [8].

### 2.3. Multilocus Sequence Typing

MLST analyses followed the guidelines on the Neisseria Multi Locus Sequence Typing website (http://pubmlst.org/neisseria). Relatedness of allelic profiles was visualized by Bionumerics 7.6 to produce minimum spanning trees. Isolates presenting a single-locus-variation in allelic profiles from a founding ST were considered as belonging to the same MLST clonal complex (CC).

### 2.4. Pulsed-Field Gel Electrophoresis (PFGE)

PFGE was performed according to the protocol described by Ribot and coworkers [15] using 15 U* SpeI* (Promega) as restriction enzyme. The CHEF DR III equipment (Bio-Rad Laboratories) was used to separate fragments, with switch times of 2 s and 17 s, and running time of 17 h at 6 V/cm. PFGE banding pattern similarities were determined with the Dice coefficient and the UPGMA (unweighted pair group method using arithmetic averages) clustering method with Biomerics 7.6. A tolerance in the band positions of 1.5% was used.

### 2.5. Identification of Azithromycin Resistance Mechanisms

Mutations at the promoter region of the gene encoding* mtr*R, the repressor of* mtr*CDE, and mutations in the four alleles of the gene* rrl*, which encode 23S rRNA, were investigated in the isolates presenting MIC ≥2 *μ*g/mL. For DNA extraction,* N. gonorrhoeae* colonies were suspended in 1 mL lysis buffer (1% triton X-100, 0.5% Tween 20, 1mM EDTA, 10mM Tris HCl, and pH 8.0) and boiled for 10 min. After centrifugation for 5 min at 10,000 rpm to remove cell debris, 1 *μ*l of the supernatant was used as the template DNA for PCR.

Promoter region of the* mtrR *gene was amplified by PCR as previously described [16]. Occurrence of mutations in* rrl* alleles was investigated by nested PCR using previously described primers [17] with modifications in the cycling conditions: all eight PCR reactions were performed at 94°C for 1 min for denaturing, 66°C for 30 seconds for annealing, and 72°C for 30 seconds for elongation, 25 cycles.

PCR products were purified with ExoSAP IT kit (Affymetrix, Santa Clara, USA). Both DNA strands of PCR products were Sanger sequenced (Macrogen Korea).

Nucleotide sequence mutation search was determined by alignment with strain FA1090 (accession numbers: NC_002946.2).

## 3. Results

### 3.1. Resistance to Azithromycin

**Figure 1:**
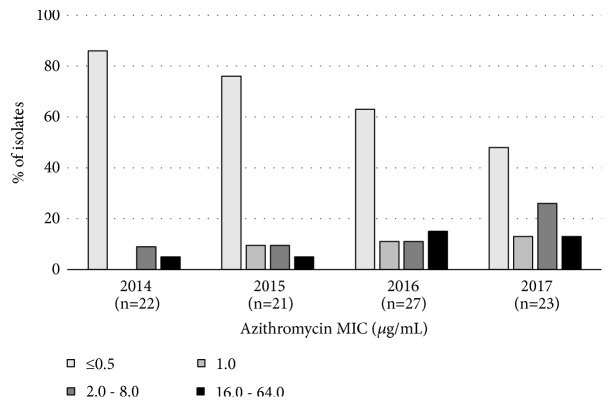
Distribution of* Neisseria gonorrhoeae* azithromycin MIC, from isolates obtained in Rio de Janeiro and characterized in this study, stratified according to year of isolation.

Throughout the study, antimicrobial susceptibility testing of all 93* N. gonorrhoeae *isolates resulted in nonsusceptibility rates for penicillin, tetracycline ciprofloxacin, and ceftriaxone of 98%, 78%, 72%, and 0%, respectively. Regarding azithromycin, 42 isolates (45%) presented MIC between ≤0.062 and <0.5 *μ*g/mL, 21 (23%) MIC 0.5 *μ*g/mL, and 30 (32%) MIC ≥1 *μ*g/mL, which would be, respectively, classified as susceptible, intermediate, or resistant to azithromycin according to EUCAST [9]. Twenty-three isolates (25%) presented MIC ≥2 *μ*g/mL, which would be recognized as non-wild-type according to CLSI [2]. The highest MIC detected was 64 *μ*g/mL for one isolate.  Figure 1 presents these results organized according to year of isolation and stratified to better demonstrate the tendency of higher MICs observed in 2016 and 2017 in comparison to the preceding years.

Further characterization regarding strain typing and azithromycin resistance mechanisms were performed for isolates presenting MIC ≥2 *μ*g/mL, which would be, according to the current literature, unanimously classified as resistant or moderately resistant [8, 11, 18, 19].

### 3.2. Typing by MLST and PFGE

**Figure 2:**
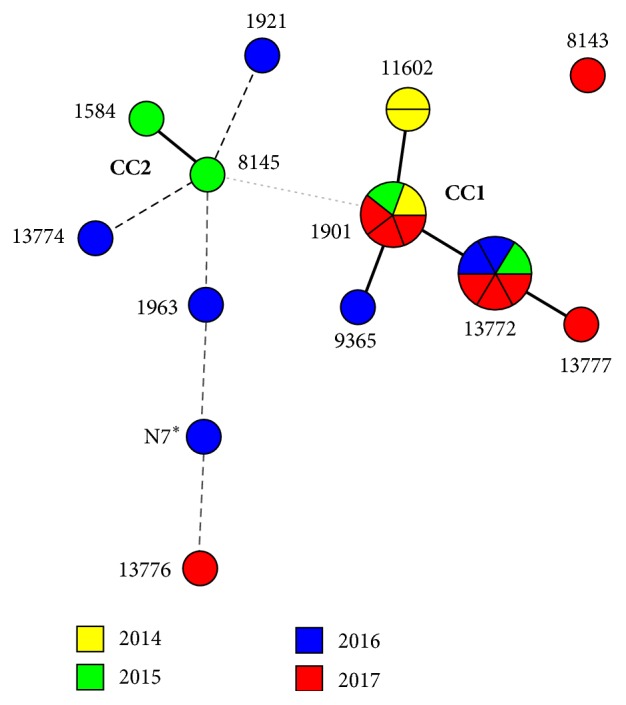
Minimum spanning tree based on MLST data of* Neisseria gonorrhoeae* isolates with MIC ≥2 *μ*g/mL obtained in Rio de Janeiro between 2014 and 2017. The colours represent the year of isolation of each isolate; solid, dashed, and dotted lines represent changes in one, two, and three alleles, respectively. ^*∗*^New undefined ST.

The 23 isolates presenting MIC ≥2 *μ*g/mL belonged to 13 different ST, five of them new (13772, 13774, 13776, 13777, and N7). As shown in Figure 2,* N. gonorrhoeae* isolates with this MIC range obtained in 2015 and 2016 were mostly nonclonally related. In contrast, most isolates with the same phenotype collected in 2017 belonged to ST1901 and its one allele variant ST13772, both part of clonal complex 1 (CC1). These two STs also concentrate isolates with higher azithromycin MIC (Table 1).

**Figure 3:**
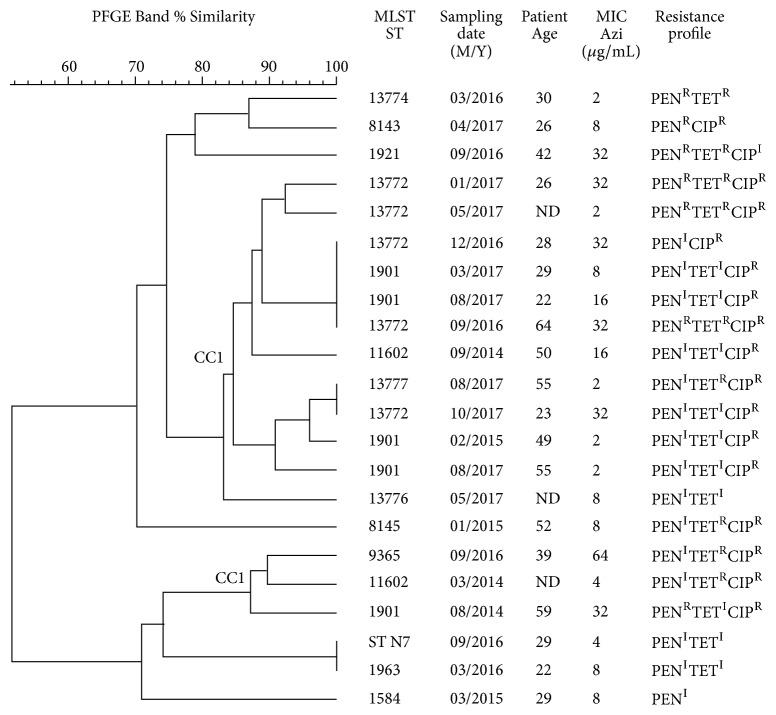
General characteristics of the azithromycin resistant isolates included in this study. M, month; Y, year; Azi, azithromycin; PEN, penicillin; TET, tetracycline; CIP, ciprofloxacin; R, resistant; I, intermediate; ND, not determined.

PFGE was performed in order to submit azithromycin resistant isolates to a second and more discriminatory typing method. Analysis of band patterns indicated that isolates belonging to CC1 (STs 13772, 1901, 11602, 13777, and 9365) grouped together with ≥ 84% similarity, whereas other isolates were more diverse.  Figure 3 shows PFGE results of these isolates combined with additional characteristics such as sampling date, antimicrobial resistance profile, and patients age.

### 3.3. Mechanisms of Azithromycin Resistance

All 23 isolates with MIC ≥2 *μ*g/mL characterized in this study had mutations at the promoter region of* mtrR* and/or at* rrl* alleles. DelA mutation at the promoter region of* mtrR* was identified in all isolates belonging to CC1. Among these isolates, those with wild type* rrl *presented azithromycin MIC 2 *μ*g/mL except for one strain with MIC 8 *μ*g/mL. Isolates with additional mutations at one to four* rrl* alleles had azithromycin MIC varying from 8 to 64 *μ*g/mL.

In contrast, isolates singletons or belonging to CC2 had a wild type promoter region of* mtrR* and mutations C2611T at four* rrl* alleles, which correlated to MIC varying from 4 to 32 *μ*g/mL. One singleton isolate was an exception. In this strain with azithromycin MIC 2 *μ*g/mL we detected substitutions, but not deletions, at the promoter region of* mtrR*, and A2059G mutations in two* rrl *alleles (Table 1).

## 4. Discussion

In this study, we evaluated the azithromycin susceptibility of 93* N. gonorrhoeae* isolates obtained in Rio de Janeiro between 2014 and 2017 and characterized the genetic diversity and main resistance mechanisms of isolates with MIC ≥2 *μ*g/mL for this antimicrobial agent.

Overall, according to the EUCAST breakpoint, the azithromycin resistant rate detected (30%) was higher than the one reported by the epidemiological study performed in seven regions of Brazil during 2015-2016 (7%) [13]. A study performed in another Brazilian city, São Paulo, with isolates collected between 2003 and 2016, reported 26% nonsusceptibility to azithromycin [20]. If we include MIC 0.5 *μ*g/mL in the calculations applied to the present collection, 55% of the isolates would be considered nonsusceptible to this drug. To the best of our knowledge, Rio de Janeiro was the first city in Brazil where isolates with azithromycin MIC ≥16 *μ*g/mL were reported [14].

Even though EUCAST determines MIC >0.5 *μ*g/mL as a breakpoint to define azithromycin resistance in* N. gonorrhoeae*, CLSI does not adopt this criterion, characterizing isolates as non-wild-type if they have MIC ≥2 *μ*g/mL. Such difference prompted us to focus on isolates with higher MICs as those with a critical phenotype. In this study, which was performed with isolates sent to our research groups by clinical laboratories without any selection criteria, analysis of strains with MIC ≥2 *μ*g/mL suggested a rising of azithromycin MIC among* N. gonorrhoeae* obtained in Rio de Janeiro over the last three years, in a phenomenon at least partly associated with the evolution of the clonal complex connected with ST1901. This ST is internationally recognized as a well succeeded clone, related to antimicrobial resistance [14, 19, 21, 22]. Interestingly, almost 50% of the isolates composing CC1 in this study belonged to new STs, especially ST13772, which was first uncovered in Rio de Janeiro in 2015. Moreover, azithromycin resistance has been additionally detected in nonclosely related strains, suggesting a diverse, polyclonal emergence of resistance to this drug, probably stimulated by the use of azithromycin to treating several community-based infections.

Generally, as expected, isolates presenting mutations only at the promoter region of* mtrR* had lower MICs than those presenting mutated* rrl *alleles. Mutations A2059G in two* rrl* alleles usually result in azithromycin MIC higher than the one observed in this work, so we confirmed all data regarding this strain. A similar unexpected association has been reported by Cole et al. [23]. Different azithromycin MICs of strains exhibiting similar resistance mechanisms may be explained by additional noninvestigated determinants, such as effect of Erm enzymes and altered identity of MtrR, L4, or L22 proteins [1, 10].

A limitation of this work is that most isolates evaluated were not obtained directly from patients but were included in the collection based on a collaboration with clinical laboratories. This may represent a bias, possibly selecting isolates from persisting or recidivist infections, since sexually transmitted infections (STI) are usually empirically treated, without microbiological analysis. In this case, it is important to note that although the use of ceftriaxone is recommended for treatment of gonorrhoea in Rio de Janeiro since 2015 [24], until 2017 there was a low availability of this drug in the decentralized healthcare network sought by patients with STI in this city. Seventy-two percent of the isolates included in this study were nonsusceptible to ciprofloxacin. Thus, the nonresponsiveness to ciprofloxacin may have influenced the composition of this collection.

The rising tendency of azithromycin resistance here reported, however, probably reflects an epidemiological event, since azithromycin resistant isolates have been obtained from patients from various age groups in different months throughout the study. As previously reported, our research group receives isolates from clinical laboratories since 2006, and our findings are product of comparison with this collection [14]. The proportion of isolates with high MICs observed during recent years is unprecedented in our collection of* N. gonorrhoeae* strains.

## 5. Conclusions

In the present work, based on 93* N. gonorrhoeae* strains obtained by clinical laboratories and sent to our research group, we identified, after 2015, a rising tendency of azithromycin resistance rates, especially considering isolates showing MIC ≥2 *μ*g/mL. These resistant isolates belonged to multiple MLST-ST; however, in 2017, a clonal complex evolutionarily related to ST1901 (CC1) drew attention, suggesting a possible expansion of this clone. Azithromycin resistant* N. gonorrhoeae *belonging to CC1 present DelA mutation at the* mtrR* promoter region associated or not with mutations in one to four* rrl *alleles, and MIC varying from 2 to 64 *μ*g/mL.

Azithromycin resistance may pose a risk for the long-term use of this drug in the combined therapy with ceftriaxone. Our data suggest that rates of azithromycin resistance in* N. gonorrhoea* in Rio de Janeiro may be higher than those reported in other Brazilian sentinel cities involved in the national surveillance program. It is very important to include Rio de Janeiro at the Brazilian GASP, in order to evaluate if these findings would be confirmed in an epidemiologically validated sampling strategy.

## Figures and Tables

**Table 1:** Description of resistance mechanisms and year of *N. gonorrhoeae* non-wild-type according to MLST ST.

CC	ST	MIC (*µ*g/mL)	Year (number of isolates)	*mtr*R Promoter/rRNA 23S (number of mutated alleles)
1	1901	32	2014 (1)	Del A/C2611T (4)
		16	2017 (1)	Del A/C2611T (4)
		8	2017 (1)	Del A/C2611T (1)
		2	2015 (1), 2017 (1)	Del A/WT
	13772	32	2016 (2), 2017 (2)	Del A/C2611T (4)
		16	2015 (1)	Del A/C2611T (4)
		2	2017 (1)	Del A/WT
	11602	8	2014 (1)	Del A/WT
		2	2014 (1)	Del A/WT
	9365	64	2016 (1)	Del A/C2611T (4)
	13777	2	2017 (1)	Del A/WT

2	8145	8	2015 (1)	WT/C2611T (4)
	1584	8	2015 (1)	WT/C2611T (4)

Sgt^*∗*^	1921	32	2016 (1)	WT/C2611T (4)
	1963	8	2016 (1)	WT/C2611T (4)
	8143	8	2017 (1)	WT/C2611T (4)
	13776	8	2017 (1)	WT/C2611T (4)
	N7	4	2016 (1)	WT/C2611T (4)
	13774	2	2016 (1)	C-34T, A-31T, A-28C/A2059G (2)

^*∗*^Singleton.

## Data Availability

Most data used to support the findings of this study are included within the article. Any other details that may interest readers are available from the corresponding author upon request.
